# Epigenetic and chromatin remodeling mechanisms across cardiomyopathies: a comprehensive review

**DOI:** 10.1186/s13072-026-00681-2

**Published:** 2026-06-10

**Authors:** Prabin Upadhyaya

**Affiliations:** 1 Division of Cardiology, Department of Medicine and Surgery, Università degli studi di Milano-Bicocca, Milan, Italy; 2 Experimental Laboratory for Genetic-Based Cardiac Arrhythmia Research, IRCCS Istituto Auxologico Italiano, Cusano Milanino, MI Italy

**Keywords:** Chromatin remodeling, Cardiomyopathy, SWI/SNF, BAF complex, NuRD complex, Polycomb, Epigenetics, Histone modifications

## Abstract

Cardiomyopathies constitute a heterogeneous group of myocardial disorders representing leading causes of heart failure and cardiovascular mortality worldwide. While genetic mutations have been extensively characterized across different cardiomyopathy subtypes, the mechanistic links between genotype and phenotype remain incompletely understood. This review synthesizes current knowledge regarding chromatin remodeling complexes and their roles in cardiac gene regulation under physiological and pathological conditions. Moreover, disease-specific chromatin remodeling patterns were examined across dilated, hypertrophic, arrhythmogenic, and restrictive cardiomyopathies, highlighting both conserved mechanisms and subtype-specific alterations. Chromatin remodeling alterations contribute significantly to cardiomyopathy pathogenesis across multiple subtypes. The reversibility of epigenetic modifications presents therapeutic opportunities not available with genetic interventions. Selective HDAC inhibitors and EZH2 antagonists show promise in preclinical models, though clinical translation requires development of cardiac-specific delivery systems. CRISPR-based epigenetic editing technologies offer future potential for precise genomic locus-specific interventions to reverse pathological transcriptional programs. Chromatin remodeling complexes including SWI/SNF (BAF), NuRD, Polycomb, ISWI, CHD, and INO80 families- modulate disease expression, progression, and phenotypic variability through epigenetic modifications and ATP-dependent chromatin remodeling. Emerging evidence demonstrates that chromatin remodelers interact dynamically with DNA methylation machinery, histone-modifying enzymes, and cardiac transcription factors to orchestrate pathological gene expression programs. Understanding these epigenetic mechanisms offers unprecedented opportunities for developing novel therapeutic strategies targeting the chromatin regulatory apparatus, potentially reversing maladaptive transcriptional programs that drive disease progression.

## Background

Cardiomyopathies represent a diverse spectrum of myocardial diseases characterized by structural and functional cardiac abnormalities that cannot be explained solely by ischemic heart disease, hypertension, valvular disease, or congenital heart disease [1]. The major cardiomyopathy subtypes: dilated cardiomyopathy (DCM), hypertrophic cardiomyopathy (HCM), arrhythmogenic cardiomyopathy (ACM), and restrictive cardiomyopathy (RCM) are traditionally classified based on morphological and hemodynamic criteria [2, 3]. In the field of cardiomyopathies, remarkable progress has been undergone in identifying causative genetic mutations in sarcomeric proteins, cytoskeletal components, ion channels and the molecular mechanisms linking genetic variants to clinical phenotypes [4]. A striking feature of inherited cardiomyopathies is their variable penetrance and phenotypic heterogeneity. Patients harboring identical pathogenic mutations often exhibit dramatically different ages of onset, disease severity, and clinical trajectories [5]. This phenotypic variability cannot be explained by DNA sequence variations alone, suggesting that additional regulatory mechanisms modulate disease expression. Epigenetic modifications, i.e. heritable changes in gene expression occurring without alterations to the underlying DNA sequence have emerged as critical modulators of cardiac gene expression and disease phenotype [6].

Chromatin remodeling, a fundamental epigenetic mechanism, involves the dynamic restructuring of chromatin architecture to regulate DNA accessibility and gene expression. This process is mediated by ATP-dependent chromatin remodeling complexes that utilize the energy derived from ATP hydrolysis to reposition, eject, or exchange nucleosomes. In mammals, chromatin remodelers are classified into four major families based on their catalytic ATPase subunits: SWI/SNF (switch/sucrose non-fermentable, also known as BAF complexes), ISWI (imitation switch), CHD (chromodomain helicase DNA-binding), and INO80 (inositol-requiring 80) [7]. Each family exhibits distinct mechanisms of action, genomic targeting specificities, and biological functions.

Recent technological advances, including chromatin immunoprecipitation sequencing (ChIP-seq), assay for transposase-accessible chromatin sequencing (ATAC-seq), and high-resolution cryo-electron microscopy, have enabled unprecedented characterization of chromatin landscapes in diseased hearts. Current knowledge regarding chromatin remodeling mechanisms in cardiomyopathies is synthesized in this review, with both disease-specific alterations and conserved pathological programs examined. The intricate interplay between chromatin remodelers, DNA methylation machinery, histone-modifying enzymes, and cardiac transcription factors is highlighted, and emerging therapeutic strategies targeting these epigenetic mechanisms are discussed.

## Methods

**Fig. 1:**
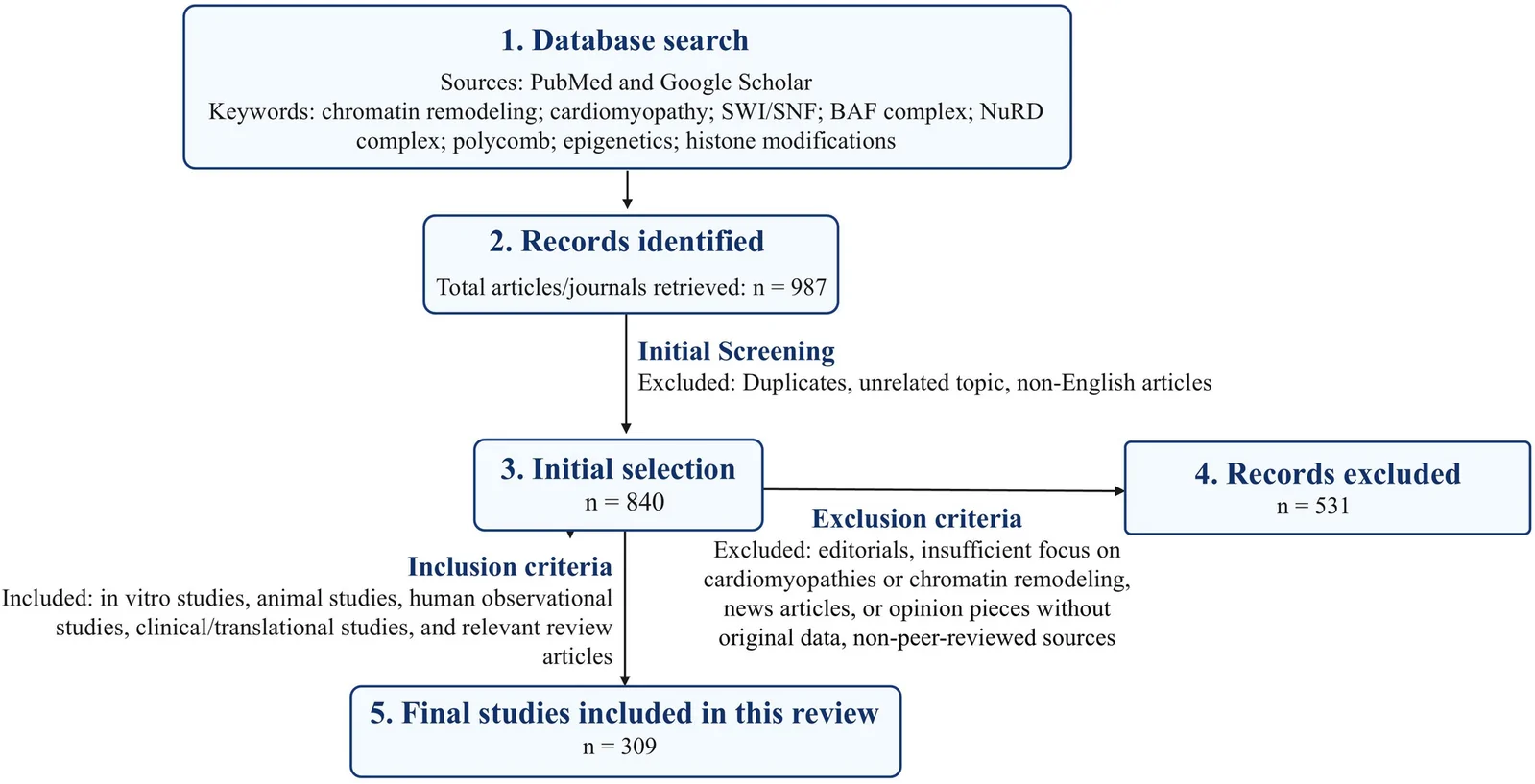
Flowchart of literature search and study selection. Flowchart summarizing the literature search and article selection process used for this review. A total of 987 records were identified through PubMed and Google Scholar using keywords related to chromatin remodeling, epigenetics, histone modifications, and cardiomyopathies. At initial selection 840 articles were processed. After screening titles, abstracts, and full-text relevance, 531 records were excluded because they were editorials, insufficient focus on cardiomyopathies or chromatin remodeling, news articles, or opinion pieces without original data, non-peer-reviewed sources. Finally, 309 peer-reviewed studies were included in the review

Literature searches were conducted in PubMed and Google Scholar using keywords (e.g. chromatin remodeling, cardiomyopathy, SWI/SNF, BAF complex, NuRD complex, polycomb, epigenetics, histone modifications, etc. alone or in combinations using Boolean operators). Related articles with epigenetic and chromatin remodeling/modifications cardiomyopathies in hypertrophic, dilated and arrhythmogenic cardiomyopathies were selected for review. Figures were designed in Biorender.com and edited in MS paint. Some figures were designed in MS PowerPoint by using public domain graphics, if and when required. A flowchart describing the literature search and selection process is provided (Fig. 1).

At the end, 309 peer-reviewed studies were included to write this review. The selected publications were further subclassified into in-vitro studies, animal model studies, mixed studies (animal + human), mixed (in-vitro + animal), human observational studies, clinical studies/trials, computational/bioinformatics reviews, and clinical guidelines. The subcategories are detailed in the Table 1.

**Table 1:** Summary of study types and evidence levels included in the review

Evidence level	Count	% of Total	Notes
In Vitro	55	17.79 %	Cell lines, primary cells, biochemical assays, iPSC-CMs used as in vitro systems
Animal Model	44	14.24%	Predominantly mouse; also zebrafish, rat, Drosophila, planarian cardiac models
Mixed (Animal + Human)	16	5.17%	Combined animal experiments with validation in human tissue/patient samples
Mixed (In Vitro + Animal)	45	14.56%	Cell culture plus animal model experiments within the same study
Human Observational	31	10.03 %	Cohort studies, case-control, GWAS, registry data, human tissue analysis
Clinical Study/Trial	1	0.32%	Regulatory approval data, interventional clinical trials, or first-in-human studies
Computational/Bioinformatic	1	0.32%	Database-driven gene/phenotype association studies without wet-lab experiments
Reviews	114	36.89%	Systematic reviews, narrative reviews, commentaries, editorials
Clinical guidelines	2	0.65%	AHA/ACC/ESC Consensus guidelines for cardiomyopathy management
TOTAL	309	100.00%	

This table summarizes the categories of studies included in the review according to their level and type of evidence

## Main body

### The Chromatin remodeling machinery: structure and function

#### SWI/SNF (BAF) chromatin remodeling complexes

**Fig. 2:**
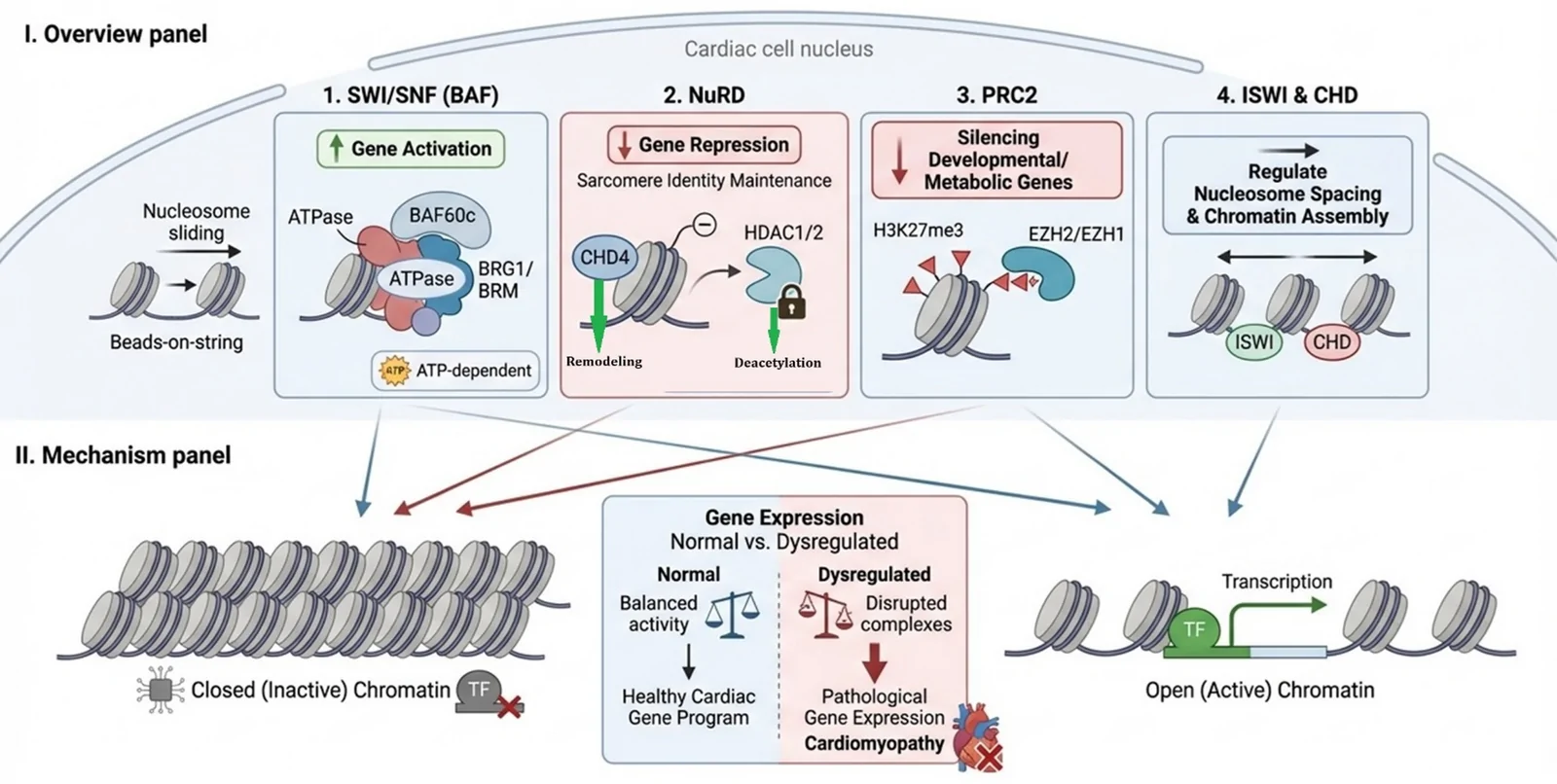
Major chromatin remodeling complex families in cardiac tissue. Four ATP-dependent chromatin remodeling families regulate cardiac gene expression. SWI/SNF (BAF) complexes, containing BRG1/BRM and cardiac-specific subunits such as BAF60c, enable nucleosome sliding and gene activation. NuRD couples CHD4 remodeling with HDAC1/2 deacetylation to enforce repression and maintain sarcomere identity. PRC2 (EZH2/EZH1) deposits H3K27me3 to silence developmental and metabolic genes. ISWI and CHD remodelers regulate nucleosome spacing and chromatin assembly. Together, these complexes transition chromatin between closed and accessible states, and their dysregulation in cardiomyopathies drives pathological transcriptional programs.

The SWI/SNF family represents one of the most extensively studied chromatin remodeling systems in cardiac biology. In mammals, the BAF (Brg1/Brahma-associated factors) complex contains 12–15 subunits assembled around a central catalytic ATPase subunit encoded by either SMARCA4 (BRG1) or SMARCA2 (BRM) [8, 9] (Fig. 2). These ATPase subunits, while highly homologous, exhibit non-redundant functions in vivo, with BRG1 being essential for embryonic development while BRM is largely dispensable [10]. Structural studies have revealed that BAF complexes exist in three major forms: canonical BAF (cBAF), polybromo-associated BAF (PBAF), and non-canonical BAF (ncBAF), each distinguished by unique subunit composition [11, 12]. In cardiac tissue, the BAF complex composition exhibits remarkable developmental stage-specificity and cell-type specificity. During early heart development, cardiac progenitors express a distinct embryonic BAF (eBAF) complex containing BAF250a (ARID1A), while differentiated cardiomyocytes express cardiomyocyte-specific subunits including BAF60c (SMARCD3) [13] (Fig. 2). BAF60c is essential for heart development and facilitates physical interactions between BRG1 and cardiogenic transcription factors including TBX5, GATA4, and NKX2-5 [14]. Biochemical purification studies have characterized cardiac-enriched BAF complexes from embryonic day 8.5 (E8.5) mouse hearts, revealing that combinatorial assembly of BAF subunits provides functional specificity resulting in unique gene expression profiles [15].

The mechanism of BAF-mediated chromatin remodeling involves ATP-dependent nucleosome sliding, ejection, and structural alteration [16]. Recent cryo-electron microscopy structures of human BAF bound to nucleosome core particles have provided atomic-level insights into the remodeling process. He et al. (2020) reported a 3.7-angstrom-resolution structure revealing that ARID1A serves as the core of the base module, stabilizing the entire complex, while SMARCA4 and SMARCB1 directly engage the nucleosome [17]. Additional structural studies by Mashtalir et al. (2020) provided insights into the endogenous human BAF complex architecture and disease-associated mutations [18]. BRG1 expression is upregulated in cardiomyocytes in response to cardiac stress and plays important roles in pathological cardiac hypertrophy [19, 20]. The dosage of BRG1 is critical for cardiogenesis, and disrupting the balance between BRG1 and cardiac transcription factors causes severe cardiac anomalies [21].

#### Nucleosome remodeling and deacetylase (NuRD) complex

The NuRD complex represents a unique chromatin remodeling entity that couples ATP-dependent chromatin remodeling with histone deacetylase activity, thereby integrating two fundamental epigenetic mechanisms [22]. The NuRD complex comprises core subunits including the ATPase CHD4 (also called Mi-2β), histone deacetylases HDAC1 and HDAC2, histone-binding proteins RbAp46 and RbAp48, metastasis-associated proteins MTA1/2/3, methyl-CpG-binding domain protein MBD3, and scaffolding proteins [23–25]. CHD4, the catalytic core of the NuRD complex, belongs to the CHD family of chromatin remodelers characterized by tandem chromodomains that recognize specific histone modifications. The dual functionality of NuRD: chromatin remodeling via CHD4 and histone deacetylation via HDAC1/2, enables coordinated regulation of chromatin accessibility and histone acetylation states. This bidirectional activity generally results in transcriptional repression, though context-dependent activation has also been observed [26, 27] (Fig. 2).

In cardiac tissue, NuRD complex activity is modulated by direct interactions with cardiac-specific transcription factors, including TBX5, TBX20, and FOG-2 (friend of GATA-2) [28]. The physical interaction between FOG-2 and components of NuRD has been shown to be essential for cardiac septation and ventricular development, with disruption of this interaction causing congenital heart defects [28, 29]. BAF250a physically interacts and functionally cooperates with NuRD complex subunits to repressively regulate chromatin structure of cardiac genes during heart development [15, 30].

#### Polycomb repressive complexes

Polycomb group (PcG) proteins form two major multiprotein complexes in mammals: Polycomb Repressive Complex 1 (PRC1) and Polycomb Repressive Complex 2 (PRC2). PRC2 catalyzes trimethylation of histone H3 at lysine 27 (H3K27me3), establishing repressive chromatin marks that silence gene expression and promote tissue-specific differentiation [31]. The core PRC2 complex comprises four essential subunits: EZH2 (enhancer of zeste homolog 2) or its paralog EZH1, which serve as the catalytic methyltransferase; EED (embryonic ectoderm development); SUZ12 (suppressor of zeste 12); and RbAp46/48 [32, 33](Fig. 2). EZH2, the primary catalytic subunit, trimethylates H3K27, creating docking sites for PRC1 complex recruitment and subsequent chromatin compaction. The H3K27me3 mark is recognized by chromodomain-containing proteins within PRC1, which catalyzes monoubiquitination of histone H2A at lysine 119 (H2AK119ub1), further stabilizing gene silencing [34]. This sequential mechanism of PRC2-mediated H3K27 trimethylation followed by PRC1 recruitment represents a canonical pathway of Polycomb-mediated gene repression (Fig. 2).

Recent studies have revealed non-canonical functions of EZH2 that extend beyond its role as a histone methyltransferase. EZH2 can function as a transcriptional co-activator through partnership with transcription factors independently of its methyltransferase activity [35]. Additionally, EZH2 can methylate non-histone proteins, including transcription factors and signaling molecules, thereby regulating their activities [36]. In cardiac contexts, EED orchestrates heart maturation through interaction with HDACs in an H3K27me3-independent manner [37].

#### ISWI and CHD family remodelers

The ISWI (imitation switch) family of chromatin remodelers is characterized by the catalytic ATPase subunits SNF2H (SMARCA5) and SNF2L (SMARCA1). These enzymes assemble with accessory subunits to form distinct complexes including NURF, ACF, CHRAC, and WICH. ISWI remodelers primarily function in nucleosome spacing and chromatin assembly, sliding nucleosomes to establish regular spacing patterns that facilitate ordered chromatin structure [38, 39].

A defining feature of ISWI complexes is their ability to sense linker DNA length through specialized domains in their C-terminal regions. The SANT (SWI3, ADA2, N-CoR, TFIIIB) and SLIDE (SANT-like ISWI) domains recognize and bind linker DNA, allowing ISWI remodelers to establish evenly spaced nucleosomal arrays [38, 40]. The CHD family, comprising nine members in mammals (CHD1-9), is distinguished by tandem chromodomains in their N-terminal regions that recognize methylated histones. CHD proteins are generally classified into three subfamilies based on domain architecture: CHD1-2 contain a DNA-binding domain; CHD3-5 (which includes CHD4 of the NuRD complex) contain plant homeodomain (PHD) fingers; and CHD6-9 contain additional specialized domains. CHD family members exhibit diverse biological functions ranging from transcriptional activation (CHD1, CHD7) to repression (CHD3-5) [41].

#### INO80 family chromatin remodelers

The INO80 family represents the fourth major class of ATP-dependent chromatin remodelers, comprising INO80 and SWR1 (SRCAP in mammals) complexes [42]. These large multisubunit assemblies are distinguished by their ability to catalyze histone variant exchange, inserting or removing variant histones such as H2A.Z. The INO80 complex contains multiple subunits and exhibits functions including nucleosome sliding, histone exchange, and DNA repair [41, 42].

While cardiac-specific functions of INO80 family remodelers remain less well-characterized compared to SWI/SNF and NuRD complexes, emerging evidence suggests important roles in cardiac development and stress responses [43]. The INO80 complex has been implicated in DNA damage repair pathways that become activated during oxidative stress and ischemic injury, conditions frequently encountered in cardiomyopathies [44, 45].

### Chromatin remodeling in cardiac development and homeostasis

#### Developmental stage-specific chromatin remodeling

Heart development represents one of the most complex organogenic processes, requiring precise temporal and spatial coordination of gene expression programs [7, 46]. Chromatin remodeling complexes play essential roles at every stage of cardiogenesis, from cardiac specification to chamber maturation [47, 48]. The SWI/SNF complex, particularly the BRG1-containing BAF complex, is indispensable for heart formation [13, 49]. Cardiac-specific deletion of Brg1 using Nkx2-5-Cre results in embryonic lethality at E10.5 (embryonic day 10.5) with severe cardiac malformations including impaired chamber septation, ventricular hypoplasia, and outflow tract defects [21, 50]. The composition of BAF complexes undergoes dynamic changes during cardiac development [51]. Embryonic stem cells express a specialized esBAF complex that is remodeled upon cardiac differentiation [52]. As cardiomyocytes differentiate, the BAF complex composition shifts to include cardiac-enriched subunits such as BAF60c (see Sect. 2.1) [53]. Inadequate expression of BAF60c impairs cardiomyocyte differentiation and disrupts expression of cardiac-specific genes including Tbx5, Nkx2-5, and Gata4 [13, 54], consistent with its role in bridging chromatin remodeling with cardiac transcriptional networks [55].

Similarly, the NuRD complex exhibits essential functions in cardiac development [56, 57]. Cardiac-conditional deletion or mutation of Chd4 results in embryonic lethality at mid-gestation with severe cardiac malformation [58, 59]. Wilczewski et al. demonstrated that CHD4 directly represses skeletal and smooth muscle myofibril isoform genes in developing cardiomyocytes [60]. In the absence of CHD4, cardiomyocytes aberrantly express skeletal muscle genes including fast skeletal α-actin (Acta1), smooth muscle myosin heavy chain (Myh11), and fast skeletal troponin components, resulting in formation of hybrid muscle cells with disrupted sarcomere structure and impaired contractile function [60].

#### Brg1/BAF complex in fetal gene program control

A critical function of the Brg1/BAF complex in adult hearts involves regulation of the myosin heavy chain (MHC) isoform switch (Fig. 4) [61]. In rodents, the adult heart predominantly expresses α-MHC (encoded by Myh6), which exhibits higher ATPase activity and contractile velocity compared to β-MHC (encoded by Myh7) [19]. During fetal development and in pathological conditions such as cardiac hypertrophy and heart failure, there is a shift from α-MHC to β-MHC expression, representing reactivation of the fetal gene program [49, 62, 63].

Brg1 plays a central role in mediating this pathological MHC switch [64]. In normal adult cardiomyocytes, Brg1 expression is downregulated, coinciding with the physiological switch from fetal β-MHC to adult α-MHC (Fig. 4) [49, 61]. However, cardiac stress induced by pressure overload, neurohormonal activation, or ischemia triggers Brg1 reactivation [65, 66]. Once reactivated, Brg1 assembles into a repressive complex with its embryonic partners, including HDACs and PARP1 (poly[ADP-ribose] polymerase 1), to suppress α-MHC and activate β-MHC expression [65, 67]. The stress-dependent assembly of the Brg1/HDAC/PARP complex represents a conserved mechanism linking hemodynamic stress to fetal gene program reactivation [68, 69]. Preventing stress-induced Brg1 expression through dominant-negative approaches or genetic deletion dramatically reduces cardiac hypertrophy, abolishes cardiac fibrosis, and reverses the pathological MHC switch in mouse models [49, 70]. Importantly, BRG1 upregulation has been documented in human hypertrophic cardiomyopathy patients, with BRG1 levels correlating with disease severity and the degree of MHC switching, suggesting clinical relevance of this mechanism [71, 72].

#### NuRD-mediated sarcomere identity maintenance

The NuRD complex plays a particularly important role in maintaining cardiac sarcomere identity by repressing expression of non-cardiac muscle gene programs. The current evidence for NuRD-mediated sarcomere identity maintenance is based primarily on genetic mouse models and mechanistic cardiomyocyte studies [60, 73]. Cardiac-specific Chd4 loss in mice derepresses skeletal and smooth muscle myofibrillar gene programs, leading to formation of hybrid sarcomeres, impaired cardiac sarcomere assembly, ventricular dysfunction, and premature death [60, 73]. These findings support a model in which CHD4/NuRD preserves cardiomyocyte identity by repressing non-cardiac muscle transcriptional programs [60, 73]. Human evidence remains more limited; however, de novo CHD4 mutations have been reported in Sifrim-Hitz-Weiss syndrome, a neurodevelopmental congenital anomaly syndrome that can include cardiac defects and skeletal muscle-related manifestations [74, 75]. Thus, while direct evidence from human cardiomyopathy tissue remains limited, available human genetic data support the clinical relevance of CHD4/NuRD dysfunction in cardiac and muscle development [74, 75].

Striated muscles (cardiac and skeletal) share similar myofibrillar sarcomeric structures but differentially express specific sarcomere components to meet their distinct contractile requirements [76]. Separation and maintenance of cardiac versus skeletal muscle identities depends critically on epigenetic repression mediated by the CHD4/NuRD complex [56, 73]. Cardiac-specific loss of CHD4 triggers aberrant expression of skeletal muscle programs, causing severe cardiomyopathy and sudden death [59, 60, 73]. Conversely, skeletal muscle-specific CHD4 deletion causes inappropriate cardiac gene expression and myopathy [73]. Recent studies have identified ZNF219 as a cardiac-specific transcription factor that recruits the CHD4/NuRD complex to skeletal muscle gene loci in cardiomyocytes [56, 77]. ZNF219 physically interacts with CHD4, and loss of either ZNF219 or CHD4 results in similar cardiac phenotypes characterized by ectopic skeletal muscle gene expression, sarcomere disorganization, fibrosis, and arrhythmias. This mechanism ensures that cardiac and skeletal muscle maintain distinct molecular identities despite their common mesodermal origin and similar contractile apparatus [78, 79].

### Chromatin remodeling in dilated cardiomyopathy

#### Overview and genetic basis

Dilated cardiomyopathy, characterized by ventricular chamber enlargement and systolic dysfunction, represents the most common cardiomyopathy subtype and a leading indication for heart transplantation [80, 81]. Unlike other cardiomyopathies, DCM exhibits remarkable genetic heterogeneity with diverse culprit gene ontologies converging on the common phenotype of a dilated and hypocontractile ventricle [82]. Over 200 genes have been associated with DCM, though strong evidence supporting pathogenicity exists for only a core subset, with the titin gene (TTN) being the most frequently implicated, accounting for 15–25% of familial DCM through truncating variants that create vulnerability to additional environmental stressors [83, 84]. Other sarcomeric genes including MYH7, TNNT2, and TPM1 collectively account for an additional 5–10% of genetic DCM cases [80]. Cytoskeletal genes such as FLNC (filamin C) and BAG3 (BCL2-associated athanogene 3) have emerged as clinically significant contributors, with FLNC truncating variants present in 1-4.5% of DCM patients and associated with an aggressive arrhythmogenic phenotype characterized by extensive myocardial fibrosis and high risk of sudden cardiac death [85–87], while BAG3 mutations, found in approximately 2% of DCM cases, typically cause early-onset disease with high penetrance in carriers over 40 years of age and a 5.1% annual incidence of adverse cardiac events [88, 89]. Nuclear envelope genes, particularly LMNA encoding lamins A and C, account for 5–8% of familial DCM and are characterized by early-onset conduction system disease, atrial and ventricular arrhythmias, and poor prognosis necessitating aggressive management including early implantable cardioverter-defibrillator placement [90, 91].

While genetic mutations in sarcomeric, cytoskeletal, and nuclear envelope proteins account for 30–40% of familial DCM cases, the mechanisms driving disease progression in both genetic and acquired forms remain incompletely understood [92, 93]. Aberrant chromatin remodeling has emerged as a significant contributor to DCM pathogenesis [94, 95].

#### BAF complex alterations in DCM

**Fig. 3:**
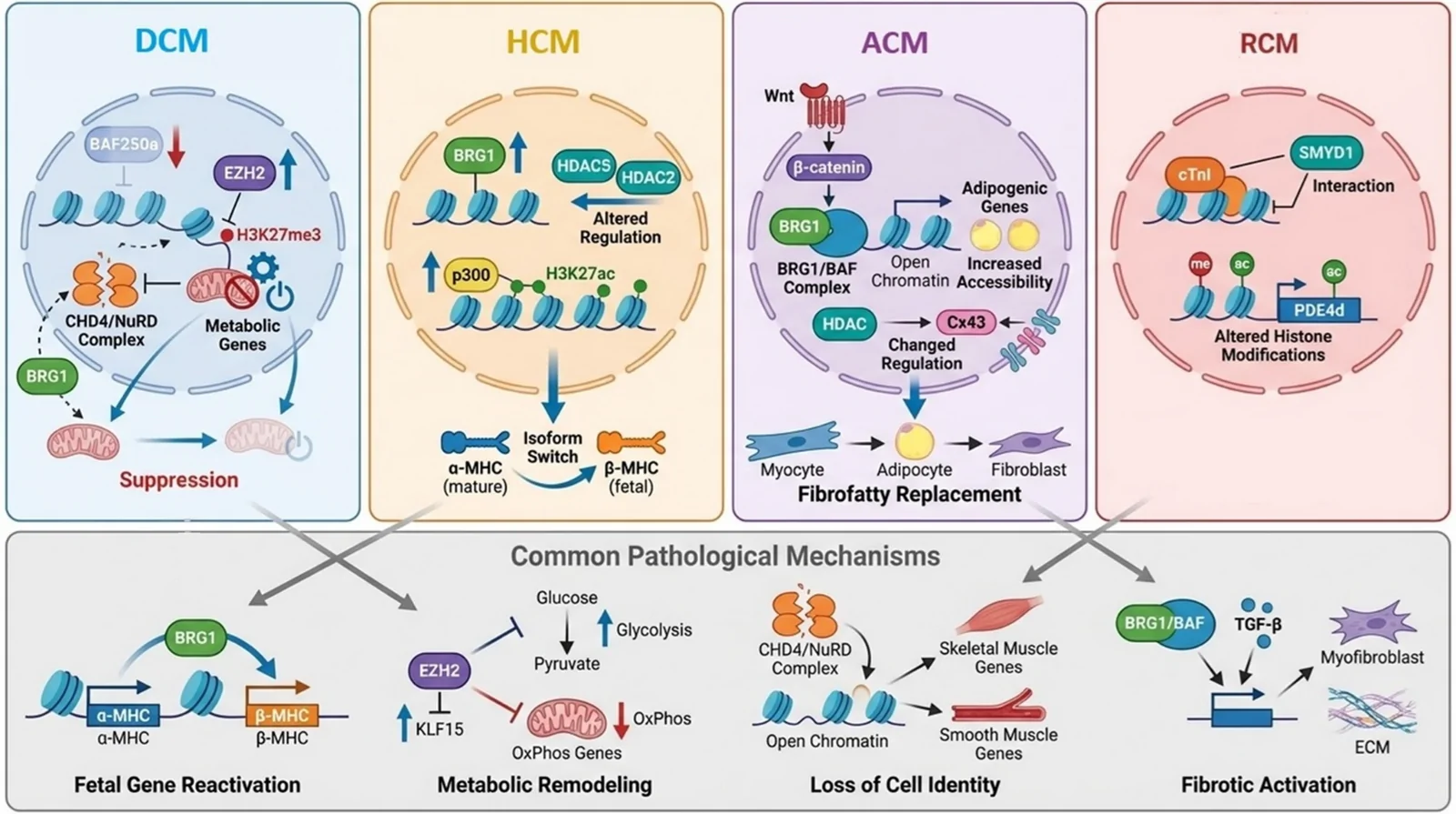
Chromatin remodeling alterations across cardiomyopathy subtypes. Cardiomyopathy subtypes display distinct chromatin remodeling signatures underlying their phenotypes. In DCM, reduced BAF250a, heightened EZH2 activity, CHD4 dysregulation, and BRG1 redistribution suppress metabolic genes. HCM involves BRG1 reactivation, HDAC5/HDAC2 imbalance, elevated p300, and H3K27ac changes driving the α- to β-MHC isoform switch. AC is characterized by Wnt/β-catenin–driven activation of BRG1/BAF, increased accessibility at adipogenic loci, and altered HDAC control of Cx43 promoting fibrofatty replacement. RCM remains poorly defined, with evidence for cTnI-SMYD1 interactions and histone changes at the PDE4d locus. Across subtypes, shared mechanisms include fetal gene reactivation (BRG1-mediated MHC switching), EZH2-driven metabolic remodeling, loss of cardiomyocyte identity via CHD4/NuRD dysfunction, and BRG1/BAF-dependent fibrotic activation through TGF-β signaling.

The BAF (BRG1/BRM-associated factor) complex, also known as the mammalian SWI/SNF complex, plays critical roles in cardiac gene regulation through ATP-dependent chromatin remodeling [49, 96]. Studies examining BAF complex function in DCM have revealed alterations in both subunit expression and complex assembly [7, 97]. In experimental models of cardiac stress and heart failure, several BAF subunits exhibit altered expression patterns [98]. BAF250a (ARID1A), a key regulatory subunit of the BAF complex, shows dynamic expression changes during cardiac remodeling [99] (Fig. 3). Reduced expression of ARID1A has been observed in failing hearts, where it correlates with altered chromatin accessibility at regulatory regions [100]. The BAF complex is essential for maintaining proper cardiac gene expression programs, and disruption of BAF complex stoichiometry can contribute to cardiac dysfunction [14, 55].

#### NuRD complex and myocardial remodeling

The nucleosome remodeling and deacetylase (NuRD) complex integrates ATP-dependent chromatin remodeling with histone deacetylation to regulate gene expression [101, 102] (Fig. 3). The NuRD complex plays important roles in cardiac development and disease [27, 103]. CHD4 (also known as Mi-2β), the catalytic chromatin remodeling component of NuRD, exhibits dynamic expression changes during cardiac stress and remodeling [104, 105]. 

Studies have shown that CHD4 expression and activity are altered during the progression of heart failure [59, 60]. The NuRD complex is involved in regulating cardiomyocyte gene expression programs, including.

genes involved in metabolism, contractility, and cell cycle regulation [73, 106]. Dysregulation of NuRD complex activity can contribute to maladaptive cardiac remodeling [73, 106]. Excessive or aberrant NuRD.

activity has been associated with cardiac dysfunction in experimental models [56, 107]. The NuRD complex regulates expression of metabolic genes critical for cardiac energy production, including components of oxidative phosphorylation and fatty acid metabolism pathways [73, 108]. Disruption of metabolic gene regulation contributes to the energy deficiency observed in failing hearts [109, 110]. The interaction between NuRD complex components and cardiac transcription factors is critical for proper gene regulation [29, 111]. FOG-2 (Friend of GATA-2, encoded by ZFPM2) is a zinc finger transcription cofactor that interacts with the NuRD complex to regulate cardiac gene expression [112, 113]. FOG-2 recruits NuRD to specific genomic loci through interactions with MTA proteins, components of the NuRD complex [114]. Alterations in FOG-2 expression or FOG-2/NuRD interactions can disrupt normal cardiac transcriptional programs [115].

#### Polycomb-mediated gene silencing in DCM

Polycomb repressive complexes (PRC1 and PRC2) are evolutionarily conserved chromatin modifying complexes that mediate gene silencing through histone modifications [116, 117]. These complexes play important roles in cardiac development and disease [37, 118] (Fig. 3). EZH2 (Enhancer of Zeste Homolog 2), the catalytic subunit of PRC2, catalyzes trimethylation of histone H3 at lysine 27 (H3K27me3), a repressive chromatin mark [119, 120]. EZH2 expression and activity are dysregulated in various forms of heart failure, including ischemic cardiomyopathy [121, 122]. EZH2 levels are elevated in failing myocardium, where increased H3K27me3 deposition occurs at genes involved in cardiac metabolism and stress response [121, 122]. Epigenetic silencing of metabolic regulators contributes to the metabolic remodeling observed in heart failure [123, 124]. Moreover, KLF15 (Krüppel-like factor 15) is a transcription factor that regulates cardiac metabolism, promoting oxidative phosphorylation and fatty acid oxidation [125, 126] (Fig. 3). EZH2-mediated epigenetic silencing can suppress KLF15 expression through H3K27 methylation [127, 128]. Reduced KLF15 expression in failing hearts contributes to metabolic reprogramming, shifting cardiac metabolism from efficient oxidative metabolism toward less efficient glycolytic pathways [126, 129, 130]. This metabolic shift contributes to energy deficiency and contractile dysfunction in DCM [131, 132] (Fig. 3).

Pharmacological inhibition of EZH2 has shown therapeutic potential in preclinical heart failure models [125, 133–135]. EZH2 inhibitors can restore expression of metabolically important genes and improve cardiac function in experimental studies [133, 136, 137]. However, EZH2 also has non-canonical functions beyond its role in PRC2, including potential transcriptional co-activator activity in certain contexts [138–142]. The context-dependent functions of EZH2 in cardiac stress responses require further investigation.

#### Chromatin accessibility and transcription factor binding

Genome-wide analysis of chromatin accessibility using ATAC-seq (Assay for Transposase-Accessible Chromatin with sequencing) has revealed global changes in chromatin structure in failing hearts [143, 144]. These studies have identified alterations in chromatin accessibility at regulatory regions of genes involved in cardiac metabolism, contractility, and extracellular matrix remodeling [143, 145]. Failing hearts exhibit widespread changes in chromatin accessibility patterns compared to non-failing controls [146]. Regions of decreased accessibility are often observed at enhancers and promoters of genes involved in normal cardiac function, while increased accessibility occurs at genes involved in pathological remodeling processes [147]. These accessibility changes reflect altered activity of chromatin remodeling complexes and correlate with corresponding gene expression changes [147]. Transcription factor binding is intimately linked to chromatin accessibility. Key cardiac transcription factors including GATA4, MEF2C, and TBX5 play essential roles in maintaining cardiac gene expression programs [148, 149]. Alterations in chromatin accessibility can affect the ability of these transcription factors to access their genomic binding sites. Reduced accessibility at cardiac transcription factor binding sites contributes to disruption of normal transcriptional programs in heart failure. Conversely, increased chromatin accessibility at binding sites for pro-fibrotic and pro-inflammatory transcription factors facilitates pathological gene expression programs [148]. Transcription factors such as AP-1 family members and SMAD proteins, which promote fibrosis and inflammatory responses, show enhanced chromatin engagement in failing hearts [150]. This reciprocal change in accessibility decreased at cardioprotective genes and increased at pathological genes reflects fundamental chromatin remodeling alterations that drive DCM progression [148].

### Chromatin remodeling in hypertrophic cardiomyopathy

#### Overview and genetic basis

Hypertrophic cardiomyopathy, characterized by asymmetric left ventricular hypertrophy in the absence of increased afterload, affects approximately 1 in 200–500 individuals and is the most common inherited cardiac disorder [151, 152]. While HCM is primarily caused by mutations in sarcomeric protein genes, most commonly MYBPC3 (cardiac myosin-binding protein C) and MYH7 (β-myosin heavy chain), epigenetic mechanisms including chromatin remodeling significantly influence disease expression and severity [153, 154]. The MYBPC3 gene accounts for approximately 40–61% of genetically identified HCM cases, making it the most prevalent causative gene in HCM [151, 155]. Most MYBPC3 mutations result in premature termination codons (PTCs) that cause nonsense-mediated decay of mutant transcripts, leading to haploinsufficiency [156, 157]. The pathogenesis of MYBPC3-related HCM involves complex mechanisms including allelic imbalance and alterations in protein quality control systems [155, 156, 158]. Multi-omics integration studies in human HCM hearts have revealed extensive chromatin remodeling alterations. Recent studies using nucleosome occupancy and methylome sequencing (NOMe-seq) combined with RNA-seq have identified differential chromatin accessibility, DNA methylation patterns, and gene expression changes in HCM patient hearts compared to controls [153, 154]. Transcriptome analyses demonstrate that HCM hearts undergo fetal gene reprogramming characterized by decreased sarcomeric and metabolic gene expression and increased extracellular matrix gene expression [153]. Chromatin accessibility analyses have identified several transcription factors, particularly SP1 and EGR1, that exhibit fetal-like binding patterns in nucleosome-depleted regions in HCM hearts [153]. Experimental inhibition of SP1 or EGR1 in HCM mouse models significantly alleviated cardiac hypertrophy and reversed fetal gene reactivation, suggesting these transcription factors as potential therapeutic targets [153].

#### BAF complex in HCM pathogenesis

The BRG1/BAF (SWI/SNF) chromatin remodeling complex exhibits altered activity in HCM hearts. BRG1 (SMARCA4), the catalytic ATPase subunit of the BAF complex, is normally downregulated in adult cardiomyocytes but becomes reactivated during pathological hypertrophy [49]. In HCM patient samples, BRG1 protein levels correlate with disease severity, wall thickness, and the degree of myosin heavy chain isoform switching [49, 72]. Mechanistic studies have demonstrated that BRG1 reactivation in HCM drives pathological hypertrophy through: (1) the MHC isoform switch (α-MHC to β-MHC, as described in Sect. 3.2), reducing contractile efficiency; (2) activation of pro-hypertrophic gene programs including natriuretic peptides (ANP, BNP); and (3) assembly of BRG1-HDAC-PARP repressive complexes that maintain cardiomyocytes in an embryonic-like state [49](Fig. 3).

Recent single-nucleus RNA-seq and ATAC-seq studies in early-stage HCM models have demonstrated activation of the SWI/SNF chromatin remodeling complex, along with IGF signaling and AP-1 transcription factors, promoting cardiomyocyte hypertrophy [150]. Other BAF complex components, including DPF3a (BAF45c), are significantly upregulated in hypertrophic hearts from HCM patients and those with aortic stenosis [159]. Upon hypertrophic stimuli, phosphorylation of DPF3a facilitates its interaction with transcriptional repressors and recruitment of BRG1 to genomic targets, initiating transcription of hypertrophic genes such as NPPA and GATA4 [159].

#### HDAC-mediated repression and cardiac hypertrophy

Histone deacetylases, while not strictly ATP-dependent chromatin remodelers, work in concert with chromatin remodeling complexes to regulate chromatin structure and gene expression in HCM [160, 161]. Class I HDACs (HDAC1, 2, 3, 8) and class IIa HDACs (HDAC4, 5, 7, 9) play critical roles in controlling hypertrophic responses [162, 163](Fig. 3). Under basal conditions, class IIa HDACs reside in the cytoplasm in a phosphorylated state. Upon dephosphorylation by calcineurin, these HDACs translocate to the nucleus where they interact with MEF2 transcription factors, repressing expression of pro-hypertrophic genes [164]. HDAC2 has been identified as particularly important in cardiac hypertrophy, with deficiency or chemical HDAC inhibition preventing re-expression of fetal genes and attenuating cardiac hypertrophy [165]. HDAC2 regulates the hypertrophic response through modulation of GSK3β activity via the Inpp5f-Akt-PDK1 pathway [165]. Class I HDACs, particularly HDAC1 and HDAC2, are required for the autophagic response that contributes to pathological cardiac remodeling [166]. Phosphorylation of HDAC2 by casein kinase-2α1 activates its pro-hypertrophic function [167]. Multiple HDAC inhibitors have demonstrated efficacy in preclinical models of cardiac hypertrophy. Trichostatin A (TSA), valproic acid (VPA), and the class I-selective inhibitor SK-7041 significantly reduce cardiac hypertrophy induced by angiotensin II infusion or aortic banding [162, 168]. These broad-spectrum HDAC inhibitors suppress ventricular growth, reduce fibrosis, preserve systolic function, and attenuate the pathological switch from adult (α-MHC) to fetal (β-MHC) myosin heavy chain isoforms [162, 168] (Fig. 3).

Remarkably, HDAC inhibition can reverse preexisting cardiac hypertrophy. Treatment with TSA in mice with established hypertrophy resulted in normalization of ventricular mass and complete restoration of ventricular function [166]. HDAC inhibitors also reduce cardiac fibrosis by blocking collagen synthesis in cardiac fibroblasts [166, 168]. However, clinical translation faces challenges. Class-selective inhibitors targeting specific HDAC isoforms may offer improved therapeutic windows by minimizing off-target effects while maintaining beneficial cardiac actions [160, 163]. Further research is needed to determine optimal HDAC isoform selectivity and dosing strategies for clinical application in HCM.

#### Polycomb regulation of cell cycle genes

While cardiac hypertrophy in HCM primarily results from cardiomyocyte enlargement rather than proliferation, Polycomb-mediated regulation of cell cycle inhibitors plays important roles in disease pathogenesis [169, 170]. EZH2 (enhancer of zeste homolog 2), the catalytic subunit of Polycomb repressive complex 2 (PRC2), trimethylates histone H3 at lysine 27 (H3K27me3), establishing repressive epigenetic marks [116, 171] (Fig. 3). EZH2 and PRC2 normally repress expression of cell cycle inhibitors including p16INK4a and p19ARF (encoded by the *Cdkn2a* locus) in embryonic and neonatal cardiomyocytes, permitting proliferation [118, 169]. He et al. (2012) demonstrated that inactivation of Ezh2 by Nkx2-5Cre in mice caused lethal congenital heart malformations, including compact myocardial hypoplasia, hypertrabeculation, and ventricular septal defect [118]. Among genes directly repressed by EZH2 were the potent cell cycle inhibitors Ink4a/b, whose upregulation was associated with decreased cardiomyocyte proliferation in Ezh2-deficient hearts [118]. In adult cardiomyocytes, PRC2 activity decreases, allowing expression of cell cycle inhibitors and establishing terminal differentiation [172]. However, EZH2 also plays critical roles in maintaining cardiac gene expression and preventing pathological remodeling in the postnatal heart. Delgado et al. (2012) showed that deletion of Ezh2 in cardiac progenitors caused postnatal myocardial pathology, including cardiomyocyte hypertrophy, increased fibrosis, and destabilized cardiac gene expression with activation of skeletal muscle genes [169]. This occurred through derepression of the homeodomain transcription factor Six1, which induced cardiomyocyte hypertrophy when ectopically expressed [169]. In HCM, altered PRC2 activity can disrupt the balance between cardiomyocyte growth and proliferation. Some studies have reported decreased EZH2 expression in hypertrophic hearts, which may derepress cell cycle inhibitors and further limit any compensatory proliferative capacity [122]. Conversely, other studies have found increased EZH2 in specific HCM models, associated with suppression of cell cycle genes [127]. These apparently contradictory findings likely reflect stage-specific and mutation-specific alterations in PRC2 function [122, 127].

Recent work has identified distinct requirements for EZH1 versus EZH2 in cardiac pathological processes. While both are catalytic subunits of PRC2, EZH1 and EZH2 exhibit partially overlapping yet distinct functions [173, 174]. EZH1, but not EZH2, is essential for neonatal heart regeneration following myocardial infarction [172]. Moreover, EZH1 overexpression improved myocardial function after injury, suggesting that selectively targeting EZH1 versus EZH2 might provide therapeutic benefits in HCM and related conditions [175].

#### CHD family remodelers in HCM

Members of the CHD (chromodomain helicase DNA-binding protein) family, particularly CHD7 and CHD8, have been implicated in cardiac development and potentially in HCM pathogenesis through their roles in regulating cardiac gene expression [176, 177]. CHD7 associates with BRG1 during neural crest development and controls expression of genes essential for cardiac outflow tract formation [177]. Mutations in *CHD7* cause CHARGE syndrome, which includes congenital heart defects [178]. While direct links between CHD7 and HCM are limited, CHD7 haploinsufficiency models exhibit cardiac abnormalities including ventricular hypertrophy and septal defects [179, 180]. CHD4, as part of the NuRD (nucleosome remodeling and deacetylase) complex, contributes to maintaining cardiac versus skeletal muscle identity [60]. In HCM hearts, dysregulation of CHD4 activity, whether arising independently or in the context of sarcomeric mutations can lead to aberrant expression of non-cardiac muscle genes. This transcriptional dysregulation may contribute to or exacerbate sarcomeric dysfunction, and notably, CHD4 dysregulation itself may drive sarcomeric abnormalities even in the absence of primary genetic mutations [73].

### Chromatin remodeling in arrhythmogenic cardiomyopathy

#### Overview and genetic basis

Arrhythmogenic cardiomyopathy (ACM), previously termed arrhythmogenic right ventricular cardiomyopathy/dysplasia (ARVC/D), is a progressive genetic cardiac disorder with a prevalence ranging from 1:1,000 to 1:5,000 [181, 182]. The disease is characterized by fibro-fatty replacement of myocardium, ventricular arrhythmias, and an increased risk of sudden cardiac death, particularly in young athletes [183, 184]. Approximately 50% of ACM patients carry genetic mutations in desmosomal genes, with plakophilin-2 (*PKP2*) being the most frequently mutated, accounting for 36–50% of genetically identified cases [185–187]. Other desmosomal genes include plakoglobin (*JUP*), desmoplakin (*DSP*), desmoglein-2 (*DSG2*), and desmocollin-2 (*DSC2*) [188, 189]. The variable penetrance and phenotypic heterogeneity observed even among patients with identical desmosomal mutations strongly suggests that epigenetic mechanisms, including chromatin remodeling, modulate disease expression [190–193]. Desmosomal dysfunction in ACM leads to complex downstream effects beyond simple structural failure. PKP2-deficient cardiomyocytes exhibit transcriptional dysregulation affecting not only desmosomal proteins but also genes encoding sarcomeric proteins, calcium handling machinery, gap junctions, and metabolic enzymes [194–196]. This broad transcriptional reprogramming implicates chromatin remodeling as a central mechanism linking primary genetic defects to the ACM phenotype.

#### Wnt/β-catenin signaling and chromatin remodeling

The most compelling evidence linking chromatin remodeling to ACM pathogenesis involves the canonical Wnt/β-catenin signaling pathway [197, 198] (Fig. 3). Plakoglobin (JUP), a component of cardiac desmosomes and a causal gene for ACM, also functions as a signaling molecule in the nucleus [199, 200]. In normal cardiomyocytes, plakoglobin competes with β-catenin for binding to TCF/LEF transcription factors, thereby suppressing Wnt target gene expression [197]. Garcia-Gras et al. (2006) demonstrated in a landmark study that suppression of desmoplakin expression in cultured atrial myocytes leads to nuclear localization of plakoglobin and a 2-fold reduction in canonical Wnt/β-catenin signaling through Tcf/Lef1 transcription factors [197]. The ensuing phenotype included increased expression of adipogenic genes (PPARγ, C/EBPα) and fibrogenic genes (collagen), along with accumulation of fat droplets, recapitulating the hallmark features of human ACM [197]. Cardiac-restricted deletion of *Dsp* in mice impaired cardiac morphogenesis, caused high embryonic lethality in the homozygous state, and in heterozygous mice, produced myocardial dysfunction and ventricular tachycardia [197].

Loss-of-function mutations in *JUP*, or sequestration of plakoglobin at disrupted desmosomes caused by other desmosomal mutations, releases plakoglobin to translocate to the nucleus where it interferes with β-catenin/Tcf transcriptional activity, leading to suppression of Wnt signaling and promoting adipogenic differentiation [198]. Lombardi et al. (2011) provided direct evidence that nuclear plakoglobin is essential for repressing Wnt signaling in cardiac progenitor cells and inducing differentiation to adipocytes, using transgenic mice overexpressing cardiac truncated plakoglobin [198]. Furthermore, genetic fate mapping experiments demonstrated that cardiac progenitor cells of the embryonic second heart field represent a cell source for excess adipocytes observed in ACM hearts [201]. Chen et al. (2014) identified activation of the Hippo pathway as an additional mechanism for suppression of canonical Wnt signaling in ACM [202]. Pathogenic activation of the Hippo kinase cascade, resulting from perturbations in intercalated discs attributable to ACM gene mutations, leads to phosphorylation and cytoplasmic retention of the transcriptional coactivator Yes-associated protein (YAP), which can sequester β-catenin and plakoglobin, resulting in reduction of both β-catenin/TCF and YAP/TEAD transcriptional activities and enhanced adipogenesis [202].

Wnt/β-catenin signaling directly regulates chromatin remodeling through recruitment of chromatin remodeling complexes. β-catenin recruits BAF/SWI-SNF chromatin remodeling complexes to Wnt target gene promoters [203, 204]. Barker et al. (2001) demonstrated that BRG1 physically interacts with β-catenin and is essential for activation of many Wnt-responsive genes [203]. The interaction between β-catenin and BRG1 enhances activity of Tcf-responsive reporter genes, and stable expression of inactive forms of BRG1 specifically inhibits expression of endogenous Tcf target genes [203]. In ACM models with disrupted desmosomal integrity, suppression of Wnt signaling would normally reduce BRG1 recruitment to Wnt target genes. However, the pathological activation of adipogenic transcription factors including PPARγ and C/EBPβ can themselves recruit chromatin remodeling complexes to adipogenic gene promoters, promoting the fibrofatty replacement characteristic of ACM [197, 201].

#### Chromatin accessibility in adipogenic conversion

The process of fibrofatty replacement in ACM involves transdifferentiation or enhanced differentiation toward adipogenic lineages, requiring extensive chromatin remodeling to silence cardiac gene programs while activating adipogenic programs [197, 205, 206]. Treatment with Wnt inhibitors or direct inhibition of chromatin remodeling activity reduces adipogenic conversion, suggesting that pathological chromatin remodeling driven by aberrant Wnt signaling is a key mechanism in ACM pathogenesis [198, 205].

Recent studies using patient-derived induced pluripotent stem cells (iPSCs) have provided insights into the chromatin landscape changes in ACM. Cardiomyocytes harboring *PKP2* mutations exhibit altered chromatin accessibility and transcriptional dysregulation affecting multiple gene networks beyond desmosomes [196, 207]. RNA sequencing analyses show that restoration of PKP2 expression leads to highly coordinated correction of PKP2-associated transcriptional networks, revealing a broad spectrum of biological perturbations behind ACM disease etiology [195, 196].

#### HDAC-mediated regulation of gap junction proteins

While not classical chromatin remodelers, HDACs collaborate with ATP-dependent chromatin remodeling complexes to regulate expression of genes critical for cardiac electrophysiology [208]. In ACM, altered expression and localization of gap junction proteins and ion channels contribute to arrhythmogenesis [209]. Studies have shown that HDAC activity modulates expression of connexin 43 (Cx43), a major gap junction protein whose downregulation is characteristic of ACM. Class I HDACs (particularly HDAC2) repress Cx43 expression through interactions with transcription factors at the *GJA1* promoter [210] (Fig. 3).

Kim et al. (2019) demonstrated that disruption of calcium homeostasis and connexin 43 hemichannel function in the right ventricle precedes overt ACM in PKP2-deficient mice, suggesting that transcriptional dysregulation of gap junction proteins is an early event in disease pathogenesis [209]. Furthermore, van Opbergen et al. (2022) showed that exercise causes arrhythmogenic remodeling of intracellular calcium dynamics in plakophilin-2-deficient hearts, highlighting the interplay between mechanical stress, transcriptional changes, and arrhythmogenesis in ACM [211].

### Chromatin remodeling in restrictive cardiomyopathy

#### Overview and genetic basis

Restrictive cardiomyopathy remains the most enigmatic cardiomyopathy subtype from an epigenetic perspective [6, 212]. Despite RCM representing a distinct clinical and pathological entity characterized by impaired ventricular filling due to increased myocardial stiffness, virtually no studies have systematically examined chromatin remodeling mechanisms specific to this condition. The rarity of RCM compared to DCM and HCM, combined with limited availability of cardiac tissue for research, has hindered investigation [213, 214]. RCM shares some genetic overlap with other cardiomyopathies, as mutations in sarcomeric protein genes including *TNNI3*, *TNNT2*, *MYH7*, and *MYBPC3* can cause RCM phenotypes in some families while causing HCM or DCM phenotypes in others [215, 216]. As we have seen in the previous sections, this mutation-independent phenotypic variability strongly suggests that epigenetic factors, including chromatin remodeling, determine which clinical phenotype emerges from a given genetic mutation [217–219].

#### Sarcomere stiffness and chromatin regulation

The hallmark feature of RCM, increased myocardial stiffness results from altered sarcomeric protein composition, excessive fibrosis, or infiltrative processes [220–222]. In genetic forms of RCM caused by troponin mutations, altered calcium sensitivity and crossbridge cycling kinetics directly increase passive myocardial stiffness. Whether chromatin remodeling mechanisms contribute to these sarcomeric alterations remains unknown, though evidence from other cardiomyopathies suggests likely involvement [6, 193, 223]. Zhao et al. (2021) provided the most detailed epigenetic study in RCM to date, examining histone modifications rather than chromatin remodeling per se. They revealed that phosphodiesterase 4d (PDE4d) is downregulated in RCM mouse hearts carrying cTnI mutations, an effect associated with enhanced acetylation of H3K4 and H3K9, and decreased trimethylation of H3K4 in the PDE4d promoter region. Moreover, they detected cTnI in cardiomyocyte nuclei and demonstrated interactions between cTnI and the histone methyltransferase SMYD1, as well as between cTnI and HDAC1 [224, 225] (Fig. 3).

These findings suggest that mutated sarcomeric proteins may directly participate in chromatin regulation by physically interacting with chromatin-modifying enzymes. If confirmed and extended, this mechanism would represent a novel paradigm linking sarcomeric dysfunction to transcriptional dysregulation through direct protein-protein interactions in the nucleus. Whether cTnI or other sarcomeric proteins also interact with ATP-dependent chromatin remodeling complexes remains to be determined [226, 227].

### Integrated chromatin remodeling networks

#### Crosstalk between chromatin remodelers and DNA methylation

**Fig. 4:**
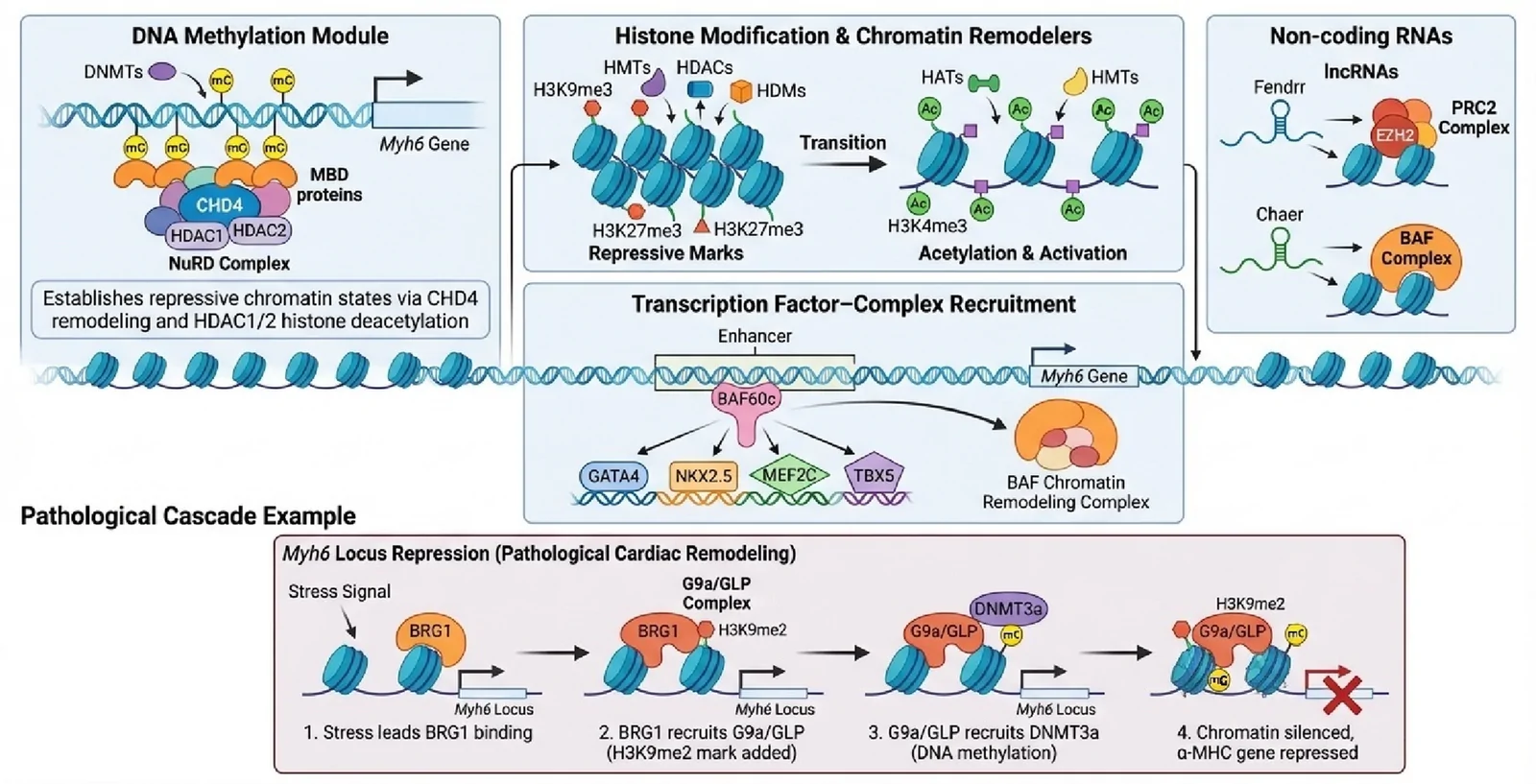
Integrated epigenetic networks in cardiac gene regulation. Chromatin remodelers act within coordinated epigenetic networks involving DNA methylation, histone modifications, transcription factors, and non-coding RNAs. DNMT-mediated DNA methylation recruits MBD proteins and the NuRD complex to establish repression. NuRD couples CHD4 remodeling with HDAC1/2 deacetylation. Cardiac TFs (GATA4, NKX2.5, MEF2C, TBX5) direct remodelers to target loci; e.g., BAF60c interacts with GATA4/TBX5 to recruit BAF to enhancers. Histone-modifying enzymes add or remove activating (H3K27ac, H3K4me3) or repressive (H3K27me3, H3K9me3) marks in concert with remodelers. lncRNAs such as Fendrr and Chaer guide PRC2 and BAF to specific loci. A key example is BRG1-dependent recruitment of G9a/GLP and subsequently DNMT3a at the Myh6 locus, depositing H3K9me2 and DNA methylation to silence α-MHC during pathological remodeling. Ac= Acetylation (Green Circle, Activating); mC- DNA Methylation (Yellow Circle, Repressive); H384me3 = Histone Methylation (Purple Square, Activating); H3K9me3, H3K27me3, H3K9me2 = Histone Methylation (Red-Orange Hexagon/ Triangle, Repressive), DNMTs = DNA Methyltransferases; MBD= Methyl-CpG Binding Domain; NuRD= Nucleosome Remodeling and Deacetylase; HATS= Histone Acetyltransferases; HDACs= Histone Deacetylases; HMTs= Histone Methyltransferases; HDMS= Histone Demethylases; BAF= BRG1/BRM-associated Factor; PRG2 = Polycomb Repressive Complex 2; IncRNAs= Long Non-coding RNAs; TFs= Transcription Factors.

Chromatin remodeling complexes do not operate in isolation but rather function within integrated epigenetic networks that include DNA methylation, histone modifications, and non-coding RNAs [228–230]. The interplay between chromatin remodelers and DNA methyltransferases (DNMTs) is particularly important in cardiomyopathies. NuRD complex components, particularly MBD3, directly recognize methylated CpG dinucleotides, providing a mechanism for targeting the complex to methylated genomic regions [231, 232](Fig. 4).

In pathological cardiac remodeling, sequential recruitment of chromatin remodeling and DNA methylation machinery establishes stable gene silencing. As described earlier, cardiac stress activates BRG1, which recruits the histone methyltransferase G9a/GLP complex. G9a deposits H3K9me2 marks, which in turn recruit DNA methyltransferase DNMT3a [233]. This cascade results in coordinated deposition of repressive histone modifications and DNA methylation at the *Myh6* locus, silencing α-MHC expression. This sequential mechanism exemplifies how chromatin remodeling initiates cascades culminating in stable epigenetic silencing [234, 235](Fig. 4).

#### Integration with histone modification machinery

The coordination between ATP-dependent chromatin remodeling and histone post-translational modifications represents a fundamental principle of epigenetic regulation [236, 237]. Chromatin

remodeling complexes both recognize specific histone modifications through reader domains and recruit histone-modifying enzymes through physical interactions (Fig. 4). For example, the chromodomains of CHD family remodelers recognize methylated H3K4, targeting these complexes to transcriptionally active regions [238, 239]. Conversely, NuRD complex recognition of methylated H3K9 and H3K27 targets it to repressed regions [239, 240] (Fig. 4). Physical interactions between chromatin remodelers and histone-modifying enzymes enable coordinated regulation. The NuRD complex, through its HDAC1/2 components, directly couples chromatin remodeling with histone deacetylation [241]. SWI/SNF complexes physically interact with histone acetyltransferases including p300 and CBP, facilitating histone acetylation at remodeled loci [242, 243]. These interactions ensure that chromatin accessibility changes are reinforced by appropriate histone modification states.

In cardiomyopathies, disruption of these coordinated mechanisms contributes to pathological transcription. For example, in HCM hearts, abnormal interactions between BRG1 and specific HDACs create regions of simultaneously altered nucleosome positioning and histone deacetylation, profoundly suppressing metabolic gene expression [168, 244].

#### Transcription factor partnerships

Chromatin remodeling complexes exhibit limited genomic targeting specificity on their own but achieve precise localization through partnerships with sequence-specific transcription factors [96, 245]. In cardiac tissue, interactions between chromatin remodelers and cardiac transcription factors including GATA4, NKX2-5, TBX5, MEF2C, and SRF enable lineage-specific gene regulation [246, 247] (Fig. 4). These transcription factors contain specialized domains that recruit chromatin remodeling complexes to cardiac gene regulatory elements. GATA4, a master cardiac transcription factor, physically interacts with BAF60c, recruiting the entire BAF complex to cardiac gene enhancers and promoters [55] (Fig. 4). This interaction is essential for activation of cardiac differentiation programs and maintenance of cardiomyocyte identity. In failing hearts, altered GATA4 expression or post-translational modifications can disrupt GATA4-BAF interactions, impairing chromatin accessibility at cardiac genes and contributing to dedifferentiation phenotypes observed in advanced cardiomyopathy [248, 249]. Similarly, MEF2 transcription factors recruit class IIa HDACs (which partner with chromatin remodeling complexes) to specific genomic loci [250, 251]. The MEF2-HDAC interaction normally represses hypertrophic gene programs, but stress-induced HDAC phosphorylation and nuclear export relieves this repression, allowing MEF2 to activate hypertrophic transcription [252–254].

#### Non-coding RNA regulation

Long non-coding RNAs (lncRNAs) and microRNAs represent additional regulatory layers that interface with chromatin remodeling machinery [253, 254]. Several cardiac-enriched lncRNAs directly interact with chromatin remodeling complexes, modulating their activity or genomic localization. For example, the lncRNA Fendrr (fetal-lethal non-coding developmental regulatory RNA) interacts with PRC2 and TrxG/MLL complexes, coordinating their recruitment to specific genomic loci during cardiac development [255–257] (Fig. 4). In pathological cardiac remodeling, stress-induced lncRNAs can aberrantly recruit chromatin remodeling complexes. The lncRNA Chaer (cardiac hypertrophy-associated epigenetic regulator) is upregulated in hypertrophic hearts and interacts with PRC2, facilitating its recruitment to metabolic gene promoters and contributing to metabolic remodeling [255, 258, 259] (Fig. 4). Similarly, the lncRNA MIAT (myocardial infarction-associated transcript) modulates chromatin accessibility in failing hearts by sequestering chromatin remodeling complex components [260, 261].

MicroRNAs also regulate chromatin remodeling indirectly by targeting mRNAs encoding chromatin remodeling complex subunits. Several microRNAs dysregulated in cardiomyopathies, including miR-1, miR-133, and miR-208, target components of BAF complexes, HDACs, and other chromatin regulators (Fig. 4). This creates complex regulatory networks where transcriptional and post-transcriptional mechanisms converge to determine chromatin states and gene expression patterns in diseased hearts [262–264].

### Therapeutic targeting of chromatin remodeling

#### HDAC inhibitors in cardiac disease

**Fig. 5:**
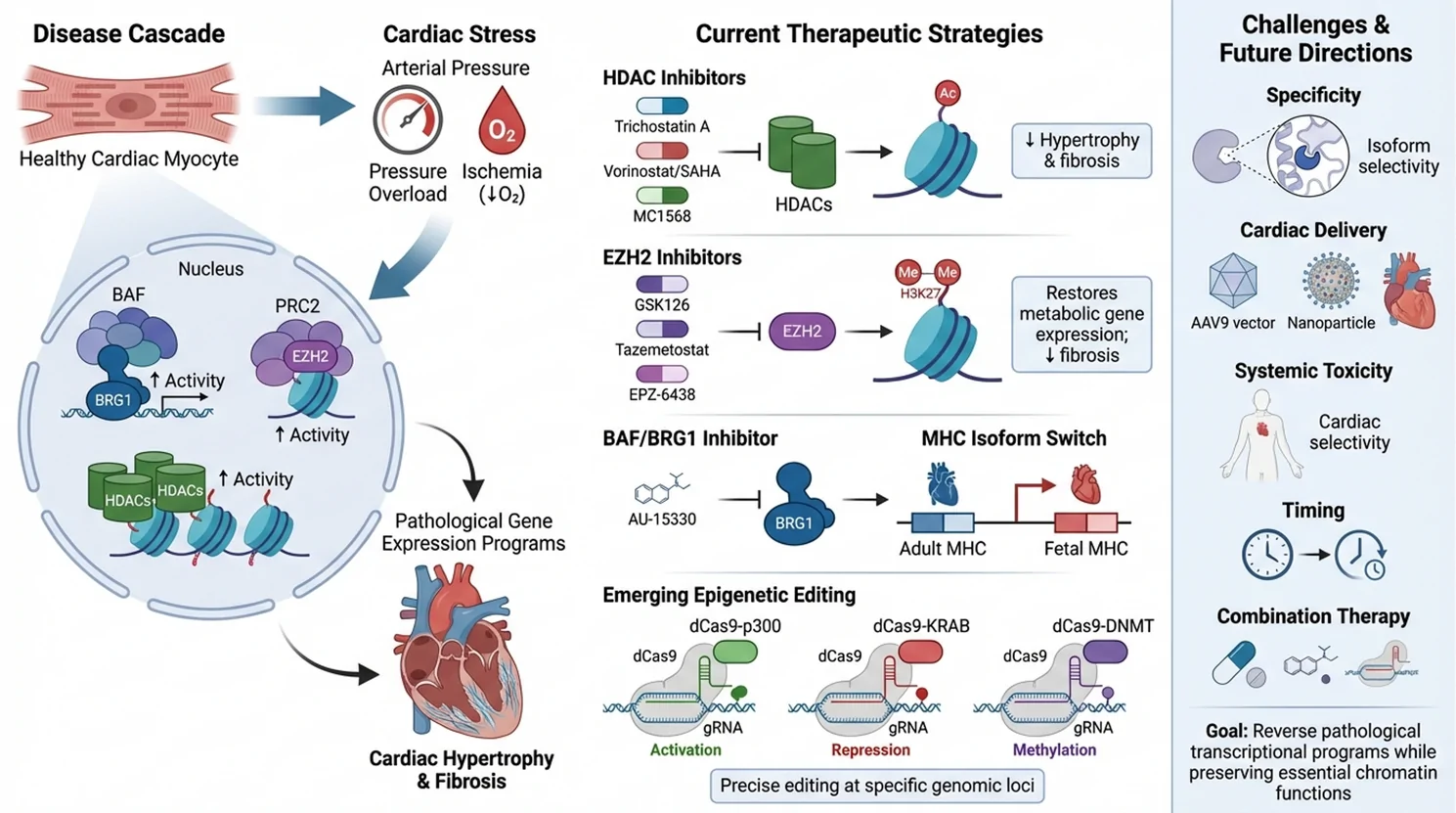
Therapeutic targeting of chromatin remodeling in cardiomyopathies. Multiple therapeutic strategies are being developed to target pathological chromatin remodeling in cardiomyopathies. Alternatively, context-dependent inhibition strategies that preferentially target disease-associated PRC2 complexes while preserving homeostatic PRC2 functions represent an aspirational goal for future drug development [32, 295].

Histone deacetylase inhibitors represent the most clinically advanced class of epigenetic therapeutics, with several compounds approved for cancer treatment [265, 266]. Their application to cardiovascular disease, particularly cardiomyopathies, has been extensively investigated in preclinical models. Pan-HDAC inhibitors including trichostatin A (TSA) and suberoylanilide hydroxamic acid (SAHA/vorinostat) demonstrate anti-hypertrophic and anti-fibrotic effects in animal models of cardiac disease [162, 166](Fig. 5). The first evidence for therapeutic potential of HDAC inhibition in cardiac disease emerged from studies showing that TSA and valproic acid could prevent cardiac hypertrophy in transgenic mice overexpressing the transcriptional repressor HOP [162]. Subsequent studies demonstrated that HDAC inhibitors suppress hypertrophy induced by β-adrenergic stimulation, angiotensin II infusion, and pressure overload [162, 166]. Importantly, HDAC inhibition not only prevents the development of hypertrophy but can also reverse established cardiac hypertrophy and improve survival in mice with pressure overload [162, 166]. The beneficial effects extend beyond hypertrophy to include reduction of cardiac fibrosis, improvement of diastolic function, and attenuation of inflammatory responses [166, 267]. Class-selective and isoform-selective HDAC inhibitors offer improved therapeutic windows compared to pan-inhibitors. Class IIa HDAC inhibitors specifically targeting HDAC4, 5, 7, and 9 show particular promise for cardiac applications. MC1568, a class IIa-selective inhibitor, reduces cardiac hypertrophy and fibrosis in pressure overload models without significant toxicity [268–270]. The selectivity of class IIa inhibitors minimizes effects on class I HDACs that are essential for normal cell proliferation and metabolism, potentially reducing adverse effects [265, 268].

Recent studies have focused on HDAC6 as a particularly attractive therapeutic target. HDAC6-selective inhibitors, including tubastatin A, demonstrate efficacy in models of heart failure with preserved ejection fraction and genetic cardiomyopathy. HDAC6 inhibition improves mitochondrial respiratory capacity in cardiomyocytes, inhibits cardiac fibroblast activation, and restores gene expression programs related to metabolism and contractility [271–273]. However, clinical translation of HDAC inhibitors for cardiomyopathies faces significant challenges. Early clinical trials of HDAC inhibitors revealed cardiovascular toxicities including QTc-interval prolongation, arrhythmias, and heart failure exacerbation in some cancer patients [274–276]. These adverse effects likely reflect the complex roles of HDACs in cardiac electrophysiology and the consequences of non-selective HDAC inhibition. Development of cardiac-targeted delivery systems or identification of context-dependent HDAC inhibition strategies may enable safer therapeutic application [277–281].

#### Small molecule modulators of ATP-dependent remodelers

Direct pharmacological targeting of ATP-dependent chromatin remodeling complexes remains challenging but represents an area of active investigation [282, 283]. Unlike HDACs, which share a conserved catalytic mechanism amenable to small molecule inhibition, chromatin remodeling ATPases exhibit structural diversity that complicates drug development. Nevertheless, several approaches are being pursued. Allosteric inhibitors targeting the BRG1/BRM ATPase have been developed, with some showing efficacy in preclinical cancer models [284, 285]. AU-15,330 is a small molecule that specifically inhibits BAF complex ATPase activity without affecting other chromatin remodeling families [286]. In cellular models of cardiac hypertrophy, AU-15,330 reduces pathological gene expression and prevents cardiomyocyte enlargement, suggesting potential therapeutic utility (Fig. 5). Similarly, AU-24,118, an orally bioavailable mSWI/SNF ATPase dual degrader has remarkable efficacy in in vitro and in vivo models [287]. However, concerns about on-target toxicity, given the essential roles of BAF complexes in normal cellular function, necessitate careful dose optimization and potentially conditional or inducible treatment strategies [18, 288]. Targeting protein-protein interactions within chromatin remodeling complexes represents an alternative approach. The interaction between BRG1 and specific accessory subunits, or between chromatin remodeling complexes and their transcription factor partners, might be more selectively disrupted without completely abolishing complex function [289, 290]. Stapled peptides and small molecules designed to disrupt specific protein interfaces are in early development stages.

#### EZH2 inhibitors and PRC2 targeting

Several selective EZH2 inhibitors have been developed for cancer treatment, with three compounds: tazemetostat, GSK126, and EPZ-6438, showing clinical efficacy [291, 292](Fig. 5). These inhibitors target the SET domain of EZH2, preventing S-adenosylmethionine binding and histone H3K27 methylation. In cardiovascular contexts, EZH2 inhibitors demonstrate beneficial effects in preclinical models of cardiac hypertrophy, fibrosis, and heart failure [135, 293] (Fig. 5). Aziz et al. (2023) demonstrated that GSK126 treatment in neonatal rat cardiac fibroblasts downregulate EZH2 expression, and reduces the downstream H3K27me3, thereby improving cardiac metabolism and cardiac fibrosis [134]. Similar beneficial effects have been observed in pressure overload-induced cardiac hypertrophy models. However, the dual roles of EZH2: as both a repressor through PRC2 and activator through non-canonical mechanisms, complicate therapeutic targeting [127]. Complete EZH2 inhibition might eliminate beneficial activator functions along with pathological repressor functions.

Selective targeting of EZH1 versus EZH2 may offer improved therapeutic windows. As mentioned earlier, EZH1 plays important roles in cardiac regeneration, suggesting that EZH2-selective inhibitors (which spare EZH1 activity) might provide cardioprotection while maintaining regenerative capacity [172, 294].

#### Targeting chromatin remodeler-transcription factor interactions

Disrupting pathological interactions between chromatin remodeling complexes and specific transcription factors offers a potentially selective therapeutic strategy. In cancer, inhibitors targeting the interaction between BRG1 and the EWS-FLI1 fusion oncoprotein show efficacy without affecting normal BRG1 functions [296, 297]. Similar approaches could be applied to cardiomyopathies. For example, the interaction between BRG1 and β-catenin drives pathological gene expression in arrhythmogenic cardiomyopathy. Small molecules or peptides designed to disrupt this specific interaction might reduce aberrant Wnt signaling and adipogenic conversion without affecting BRG1’s essential functions in other cellular processes [197, 203]. Similarly, disrupting pathological FOG-2/NuRD interactions in failing hearts might restore appropriate transcriptional programs.

#### Epigenetic editing and future directions

CRISPR-based epigenetic editing technologies offer unprecedented precision for modifying chromatin states at specific genomic loci [298, 299]. Catalytically dead Cas9 (dCas9) fused to chromatin remodeling domains, histone-modifying enzymes, or transcriptional effectors can be targeted to specific genes to alter their expression without changing DNA sequences. dCas9-p300 fusions activate target genes by depositing H3K27ac marks, while dCas9-KRAB or dCas9-DNMT fusions repress genes through heterochromatin formation or DNA methylation [299, 300] (Fig. 5). In cardiac research, epigenetic editors have been used to reverse pathological gene expression patterns in cultured cardiomyocytes and in vivo models. For example, targeting dCas9-p300 to metabolic gene promoters in failing cardiomyocytes restores their expression and improves contractile function [301]. Conversely, targeting dCas9-KRAB to fibrotic gene promoters reduces collagen expression and attenuates fibrosis. While still experimental, these technologies demonstrate proof-of-concept for precise epigenetic interventions [298, 302].

Translation of epigenetic editing to clinical applications faces substantial hurdles including delivery challenges, potential off-target effects, and immunogenicity concerns [303, 304]. Adeno-associated viruses (AAVs) with cardiac tropism can deliver epigenetic editors to cardiomyocytes, but achieving sufficient transduction efficiency in failing hearts remains difficult [305–307]. Lipid nanoparticles and engineered exosomes represent alternative delivery vehicles under investigation [308, 309].

## Conclusions and future perspectives

Chromatin remodeling has emerged as a critical regulatory mechanism in cardiomyopathy pathogenesis, influencing disease onset, progression, and phenotypic expression. The four major families of ATP-dependent chromatin remodelers, SWI/SNF, NuRD, Polycomb, and ISWI/CHD exhibit distinct yet overlapping functions in cardiac gene regulation under physiological and pathological conditions. Across cardiomyopathy subtypes, altered chromatin remodeling contributes to pathological transcriptional programs including metabolic remodeling, fetal gene reactivation, fibrotic responses, and loss of cellular identity. Several unifying themes emerge from this review. First, chromatin remodeling complexes function within integrated epigenetic networks that include DNA methylation, histone modifications, and non-coding RNAs. Understanding these interconnections is essential for developing effective therapeutic strategies. Second, chromatin remodelers achieve specificity through partnerships with transcription factors and chromatin-binding proteins, enabling precise genomic targeting despite limited intrinsic sequence specificity. Third, many chromatin remodeling alterations observed in cardiomyopathies represent stress-responsive adaptive programs that become maladaptive with sustained activation.

Significant knowledge gaps remain, particularly regarding chromatin remodeling mechanisms in arrhythmogenic and restrictive cardiomyopathies. Systematic application of contemporary epigenomic technologies including ATAC-seq, CUT&RUN, single-cell multi-omics, and spatial epigenomics will be essential to comprehensively map chromatin landscapes across all cardiomyopathy subtypes. These studies should examine multiple time points during disease progression to identify critical transition points and optimal intervention windows. From a therapeutic perspective, the reversibility of chromatin remodeling represents both opportunity and challenge. Unlike genetic mutations, chromatin states can be pharmacologically modified, offering hope for reversing pathological transcriptional programs. However, the pleiotropic functions of chromatin remodeling complexes and their essential roles in normal physiology necessitate highly selective targeting strategies to avoid toxicity. Development of cardiac-specific, isoform-selective, or context-dependent chromatin remodeling modulators represents a critical priority.

The convergence of chromatin biology, cardiovascular medicine, and therapeutic innovation positions chromatin remodeling as a central frontier in cardiomyopathy research. As our understanding deepens and technologies advance, chromatin-targeted therapies may transform management of these devastating cardiac diseases, offering hope for improved outcomes and quality of life for affected patients.

## Data Availability

Not applicable.

## Abbreviations

AAV: Adeno-associated virus
AAVs: Adeno-associated viruses
ACF: ATP-utilizing chromatin assembly and remodeling factor
ACM: Arrhythmogenic cardiomyopathy
ACTC1: Actin alpha cardiac muscle 1
AMPK: AMP-activated protein kinase
ANP: Atrial natriuretic peptide
AP: 1-Activator protein 1
ARID1A: AT-rich interaction domain 1 A
ARID1B: AT-rich interaction domain 1B
ARID2: AT-rich interaction domain 2
ARVC/D: Arrhythmogenic right ventricular cardiomyopathy/dysplasia
ATAC: seq-Assay for transposase-accessible chromatin using sequencing
ATP: Adenosine triphosphate
ATPase: Adenosine triphosphatase
BAF: BRG1/BRM-associated factor
BAF45c: DPF3a
BAF60c: SMARCD3
BAG3: BCL2-associated athanogene 3
BNP: B-type natriuretic peptide
BRG1: Brahma-related gene 1 (SMARCA4)
BRM: Brahma (SMARCA2)
cBAF: Canonical BAF
CHD: Chromodomain helicase DNA-binding
CHD1: 9-Chromodomain helicase DNA-binding proteins 1-9
CHD4: Chromodomain helicase DNA-binding protein 4
CHD7: Chromodomain helicase DNA-binding protein 7
CHD8: Chromodomain helicase DNA-binding protein 8
ChIP: seq-Chromatin immunoprecipitation sequencing
CHRAC: Chromatin accessibility complex
CRISPR: Clustered regularly interspaced short palindromic repeats
DCM: Dilated cardiomyopathy
DNA: Deoxyribonucleic acid
DNMT: DNA methyltransferase
DPF3a: Double PHD fingers 3a
DSC2: Desmocollin 2
DSG2: Desmoglein 2
DSP: Desmoplakin
eBAF: Embryonic BAF
ECM: Extracellular matrix
EED: Embryonic ectoderm development
EGR1: Early growth response 1
ERK: Extracellular signal-regulated kinase
ESC: European Society of Cardiology
esBAF: Embryonic stem cell BAF
EZH1: Enhancer of zeste homolog 1
EZH2: Enhancer of zeste homolog 2
FLNC: Filamin C
FOG: 2-Friend of GATA-2
GATA4: GATA binding protein 4
GATA5: GATA binding protein 5
GATA6: GATA binding protein 6
GJA1: Gap junction alpha 1 (connexin 43)
GSK3β: Glycogen synthase kinase 3 beta
H2A.Z: Histone H2A variant Z
H3K27ac: Histone H3 lysine 27 acetylation
H3K27me3: Histone H3 lysine 27 trimethylation
H3K4me3: Histone H3 lysine 4 trimethylation
H3K9me3: Histone H3 lysine 9 trimethylation
HAT: Histone acetyltransferase
HCM: Hypertrophic cardiomyopathy
HDAC: Histone deacetylase
HDAC1, 2, 4, 5, 7, 9: Histone deacetylases 1, 2, 4, 5, 7, 9
Hippo: Hippo signaling pathway
IGF: Insulin-like growth factor
INO80: Inositol-requiring 80
Inpp5f: Inositol polyphosphate-5-phosphatase F
ISWI: Imitation switch
JUP: Junction plakoglobin
lncRNA: Long non-coding RNA
LNP: Lipid nanoparticle
LV: Left ventricular
MAPK: Mitogen-activated protein kinase
MBD3: Methyl-CpG-binding domain protein 3
MEF2: Myocyte enhancer factor 2
MHC: Myosin heavy chain
MIAT: Myocardial infarction-associated transcript
miRNA: MicroRNA
MTA: Metastasis-associated protein
MYBPC3: Myosin-binding protein C3
MYH6: Myosin heavy chain 6 (α-MHC)
MYH7: Myosin heavy chain 7 (β-MHC)
MYL2: Myosin light chain 2
MYL3: Myosin light chain 3
nBAF: Neuronal BAF
ncBAF: Non-canonical BAF
NFAT: Nuclear factor of activated T-cells
NKX2: 5-NK2 homeobox 5
NMD: Nonsense-mediated decay
NOMe: seq-Nucleosome occupancy and methylome sequencing
npBAF: Non-polybromo BAF
NPPA: Natriuretic peptide A
NURF: Nucleosome remodeling factor
NuRD: Nucleosome remodeling and deacetylase
PBAF: Polybromo-associated BAF
PDK1: Phosphoinositide-dependent kinase 1
PHD: Plant homeodomain
PI3K: Phosphoinositide 3-kinase
PKP2: Plakophilin 2
PRC1: Polycomb repressive complex 1
PRC2: Polycomb repressive complex 2
PTC: Premature termination codon
RbAp46: Retinoblastoma-associated protein 46
RbAp48: Retinoblastoma-associated protein 48
RCM: Restrictive cardiomyopathy
RNA: Ribonucleic acid
RNA: seq-RNA sequencing
RV: Right ventricular
SANT: SWI3, ADA2, N-CoR, TFIIIB domain
scRNA: seq-Single-cell RNA sequencing
SLIDE: SANT-like ISWI domain
SMARCA1: SNF2L
SMARCA2: BRM
SMARCA4: BRG1
SMARCA5: SNF2H
SMARCD3: BAF60c
SNF2H: Sucrose nonfermenting protein 2 homolog
SNF2L: Sucrose nonfermenting protein 2-like
snRNA: seq-Single-nucleus RNA sequencing
SP1: Specificity protein 1
SRCAP: SNF2-related CREBBP activator protein
SUZ12: Suppressor of zeste 12
SWI/SNF: Switch/Sucrose non-fermentable
SWR1: SWI2/SNF2-related 1
TAZ: Transcriptional coactivator with PDZ-binding motif
TBX5: T-box transcription factor 5
TBX20: T-box transcription factor 20
TCF/LEF: T-cell factor/lymphoid enhancer-binding factor
TEAD: TEA domain transcription factor
TGF: β-Transforming growth factor beta
TNNI3: Troponin I3
TNNT2: Troponin T2
TPM1: Tropomyosin 1
TSA: Trichostatin A
TTN: Titin
VPA: Valproic acid
WICH: WSTF-ISWI chromatin remodeling complex
Wnt: Wingless-related integration site
WWTR1: WW domain-containing transcription regulator 1
YAP: Yes-associated protein
α: MHC-Alpha-myosin heavy chain (MYH6)
β: MHC-Beta-myosin heavy chain (MYH7)
